# 2,2′-[Pyridine-2,6-diylbis(carbonyl­hydrazono)]dipropanoic acid *N*,*N*-dimethyl­formamide disolvate

**DOI:** 10.1107/S160053680903997X

**Published:** 2009-10-07

**Authors:** Yanling Qiao, Jichun Cui, Longhua Ding, Handong Yin

**Affiliations:** a College of Chemistry and Chemical Engineering, Liaocheng University, Shandong 252059, People’s Republic of China

## Abstract

The complete molecule of the title compound, C_13_H_13_N_5_O_6_·2C_3_H_7_NO, is generated by crystallographic twofold rotation with an N and a C atom lying on the axis. The structure is stabilized by inter­molecular O—H⋯O hydrogen bonds.

## Related literature

For the synthesis and structures of some organotin(IV) complexes of related tridentate hydrazone ligands see: Yin *et al.* (2008 ▶).
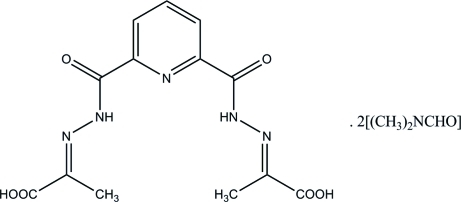


## Experimental

### 

#### Crystal data

C_13_H_13_N_5_O_6_·2C_3_H_7_NO
*M* *_r_* = 481.48
Monoclinic, 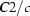

*a* = 19.5743 (17) Å
*b* = 10.4041 (11) Å
*c* = 11.7924 (12) Å
β = 107.684 (1)°
*V* = 2288.1 (4) Å^3^
*Z* = 4
Mo *K*α radiation
μ = 0.11 mm^−1^
*T* = 298 K
0.40 × 0.39 × 0.17 mm

#### Data collection

Siemens CCD area-detector diffractometer
Absorption correction: multi-scan (*SADABS*; Sheldrick, 1996 ▶) *T*_min_ = 0.957, *T*_max_ = 0.981
5528 measured reflections
2017 independent reflections
1102 reflections with *I* > 2σ(*I*)
*R*_int_ = 0.041

#### Refinement

*R*[*F* ^2^ > 2σ(*F* ^2^)] = 0.045
*wR*(*F* ^2^) = 0.137
*S* = 1.00
2017 reflections
158 parameters
H-atom parameters constrained
Δρ_max_ = 0.20 e Å^−3^
Δρ_min_ = −0.14 e Å^−3^

### 

Data collection: *SMART* (Siemens, 1996 ▶); cell refinement: *SAINT* (Siemens, 1996 ▶); data reduction: *SAINT*; program(s) used to solve structure: *SHELXS97* (Sheldrick, 2008 ▶); program(s) used to refine structure: *SHELXL97* (Sheldrick, 2008 ▶); molecular graphics: *SHELXTL* (Sheldrick, 2008 ▶); software used to prepare material for publication: *SHELXTL*.

## Supplementary Material

Crystal structure: contains datablocks I, global. DOI: 10.1107/S160053680903997X/sj2660sup1.cif

Structure factors: contains datablocks I. DOI: 10.1107/S160053680903997X/sj2660Isup2.hkl

Additional supplementary materials:  crystallographic information; 3D view; checkCIF report

## Figures and Tables

**Table 1:** Hydrogen-bond geometry (Å, °)

*D*—H⋯*A*	*D*—H	H⋯*A*	*D*⋯*A*	*D*—H⋯*A*
O3—H3⋯O4	0.82	1.75	2.574 (3)	178

**Fig. 1.**
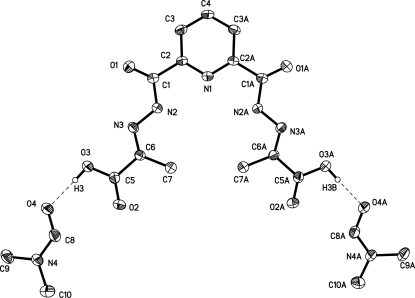
The structure of the title compound, with 50% probability displacement ellipsoids and hydrogen bonds drawn as dashed lines. H atoms not involved in hydrogen bonding have been omitted for clarity.

## supplementary crystallographic information

### Comment

Recently, we have reported some organotin(IV) complexes with hydrazone ligands (Yin *et al.*,  2008). As an extension of our work on the structural characterization of hydrazone compounds, the title compound, (I), is reported here.

The two halves of  the main molecule are symmetrically related, Fig. 1,  with the N1, C4 and H4 atoms of the pyridine ring lying on the two-fold axis to form a helical species. The N3═C6 bond length of 1.283 (3) Å (Table 1) conforms to the value for a double bond, while the N2—C1 [1.361 (3) Å] and N2—N3 [1.369 (3) Å] bonds are intermediate between a double bond and a single bond because of conjugation effects in the molecule.

### Experimental

Compound (I) was synthesized by the reaction of pyridine-2,6-dihydrazide (5 mmol) with pyruvic acid (10 mmol). Single crystals of (I) suitable for X-ray diffraction were obtained by slow evaporation of an N,N-dimethylformamide solution.

### Refinement

The H atoms were positioned geometrically, with methyl C—H distances of 0.96 Å, aromatic and aldehydic C—H distances of 0.93 Å, N—H distances of 0.86 Å and O—H distances of 0.82 Å, and refined as riding on their parent atoms, with *U*_iso_(H) = 1.2 *U*_eq_(C, N) or 1.5 *U*_eq_(C, O) for the methyl groups.

### Crystal data

**Table:**

C_13_H_13_N_5_O_6_·2C_3_H_7_NO	*F*(000) = 1016
*M**_r_* = 481.48	*D*_x_ = 1.398 Mg m^−^^3^
Monoclinic, *C*2/*c*	Mo *K*α radiation, λ = 0.71073 Å
Hall symbol: -C 2yc	Cell parameters from 1027 reflections
*a* = 19.5743 (17) Å	θ = 2.2–21.9°
*b* = 10.4041 (11) Å	µ = 0.11 mm^−^^1^
*c* = 11.7924 (12) Å	*T* = 298 K
β = 107.684 (1)°	Block, colorless
*V* = 2288.1 (4) Å^3^	0.40 × 0.39 × 0.17 mm
*Z* = 4	

### Data collection

**Table:**

Siemens CCD area-detector diffractometer	2017 independent reflections
Radiation source: fine-focus sealed tube	1102 reflections with *I* > 2σ(*I*)
graphite	*R*_int_ = 0.041
φ and ω scans	θ_max_ = 25.0°, θ_min_ = 2.2°
Absorption correction: multi-scan (*SADABS*; Sheldrick, 1996)	*h* = −23→23
*T*_min_ = 0.957, *T*_max_ = 0.981	*k* = −6→12
5528 measured reflections	*l* = −12→14

### Refinement

**Table:**

Refinement on *F*^2^	Primary atom site location: structure-invariant direct methods
Least-squares matrix: full	Secondary atom site location: difference Fourier map
*R*[*F*^2^ > 2σ(*F*^2^)] = 0.045	Hydrogen site location: inferred from neighbouring sites
*wR*(*F*^2^) = 0.137	H-atom parameters constrained
*S* = 1.00	*w* = 1/[σ^2^(*F*_o_^2^) + (0.0581*P*)^2^ + 0.8585*P*] where *P* = (*F*_o_^2^ + 2*F*_c_^2^)/3
2017 reflections	(Δ/σ)_max_ = 0.004
158 parameters	Δρ_max_ = 0.20 e Å^−^^3^
0 restraints	Δρ_min_ = −0.14 e Å^−^^3^

### Fractional atomic coordinates and isotropic or equivalent isotropic displacement parameters (Å^2^)

**Table:**

	*x*	*y*	*z*	*U*_iso_*/*U*_eq_	
N1	0.5000	0.2225 (3)	0.7500	0.0370 (8)	
N2	0.56173 (12)	0.3597 (2)	0.6173 (2)	0.0440 (6)	
H2	0.5454	0.3894	0.6721	0.053*	
N3	0.58981 (11)	0.4428 (2)	0.55325 (18)	0.0414 (6)	
N4	0.71491 (12)	1.0005 (2)	0.3217 (2)	0.0492 (7)	
O1	0.58033 (11)	0.18075 (19)	0.52026 (18)	0.0608 (6)	
O2	0.62171 (12)	0.76940 (19)	0.54017 (18)	0.0615 (6)	
O3	0.64206 (11)	0.60873 (17)	0.43031 (16)	0.0539 (6)	
H3	0.6570	0.6669	0.3972	0.081*	
O4	0.69084 (11)	0.78824 (19)	0.32604 (17)	0.0546 (6)	
C1	0.55919 (15)	0.2310 (3)	0.5960 (2)	0.0414 (7)	
C2	0.52726 (14)	0.1563 (2)	0.6765 (2)	0.0365 (7)	
C3	0.52768 (15)	0.0235 (3)	0.6730 (2)	0.0472 (7)	
H3A	0.5462	−0.0199	0.6199	0.057*	
C4	0.5000	−0.0427 (4)	0.7500	0.0550 (12)	
H4	0.5000	−0.1320	0.7500	0.066*	
C5	0.62039 (14)	0.6566 (3)	0.5162 (2)	0.0417 (7)	
C6	0.59176 (14)	0.5610 (3)	0.5853 (2)	0.0394 (7)	
C7	0.56634 (16)	0.6134 (3)	0.6825 (3)	0.0569 (9)	
H7A	0.5151	0.6058	0.6610	0.085*	
H7B	0.5797	0.7023	0.6950	0.085*	
H7C	0.5878	0.5660	0.7544	0.085*	
C8	0.69523 (15)	0.8983 (3)	0.3682 (3)	0.0502 (8)	
H8	0.6836	0.9088	0.4385	0.060*	
C9	0.73224 (17)	0.9912 (3)	0.2111 (3)	0.0645 (10)	
H9A	0.7245	0.9046	0.1818	0.097*	
H9B	0.7816	1.0141	0.2246	0.097*	
H9C	0.7021	1.0486	0.1536	0.097*	
C10	0.7204 (2)	1.1255 (3)	0.3783 (3)	0.0787 (11)	
H10A	0.6987	1.1220	0.4412	0.118*	
H10B	0.6961	1.1884	0.3206	0.118*	
H10C	0.7700	1.1488	0.4106	0.118*	

### Atomic displacement parameters (Å^2^)

**Table:**

	*U*^11^	*U*^22^	*U*^33^	*U*^12^	*U*^13^	*U*^23^
N1	0.0357 (18)	0.0301 (17)	0.0426 (18)	0.000	0.0078 (15)	0.000
N2	0.0566 (16)	0.0331 (13)	0.0493 (14)	−0.0003 (12)	0.0264 (12)	0.0034 (11)
N3	0.0420 (14)	0.0390 (14)	0.0456 (14)	0.0005 (12)	0.0170 (11)	0.0080 (12)
N4	0.0583 (16)	0.0443 (14)	0.0490 (15)	−0.0039 (13)	0.0222 (13)	0.0069 (13)
O1	0.0845 (16)	0.0466 (13)	0.0638 (14)	−0.0028 (12)	0.0410 (12)	−0.0082 (11)
O2	0.0899 (17)	0.0351 (12)	0.0710 (15)	−0.0122 (12)	0.0419 (13)	−0.0040 (11)
O3	0.0756 (14)	0.0412 (12)	0.0559 (13)	−0.0021 (11)	0.0360 (11)	0.0046 (10)
O4	0.0693 (14)	0.0457 (13)	0.0542 (13)	−0.0067 (11)	0.0268 (11)	0.0046 (11)
C1	0.0444 (17)	0.0342 (16)	0.0452 (17)	0.0023 (14)	0.0129 (14)	−0.0011 (14)
C2	0.0395 (16)	0.0276 (15)	0.0402 (16)	0.0001 (13)	0.0089 (13)	−0.0028 (13)
C3	0.0542 (19)	0.0338 (16)	0.0558 (19)	0.0004 (15)	0.0202 (15)	−0.0081 (15)
C4	0.075 (3)	0.025 (2)	0.073 (3)	0.000	0.034 (3)	0.000
C5	0.0407 (17)	0.0444 (19)	0.0420 (17)	−0.0004 (15)	0.0154 (14)	0.0015 (14)
C6	0.0404 (17)	0.0343 (16)	0.0459 (16)	0.0010 (14)	0.0169 (13)	0.0038 (14)
C7	0.075 (2)	0.0429 (18)	0.065 (2)	−0.0003 (17)	0.0401 (18)	−0.0006 (16)
C8	0.055 (2)	0.054 (2)	0.0442 (17)	−0.0059 (17)	0.0193 (15)	0.0034 (16)
C9	0.081 (2)	0.062 (2)	0.057 (2)	−0.0082 (19)	0.0322 (18)	0.0150 (17)
C10	0.108 (3)	0.048 (2)	0.093 (3)	−0.009 (2)	0.049 (2)	−0.008 (2)

### Geometric parameters (Å, °)

**Table:**

N1—C2^i^	1.339 (3)	C3—C4	1.375 (3)
N1—C2	1.339 (3)	C3—H3A	0.9300
N2—C1	1.361 (3)	C4—C3^i^	1.375 (3)
N2—N3	1.369 (3)	C4—H4	0.9300
N2—H2	0.8600	C5—C6	1.499 (4)
N3—C6	1.283 (3)	C6—C7	1.485 (4)
N4—C8	1.308 (3)	C7—H7A	0.9600
N4—C9	1.447 (3)	C7—H7B	0.9600
N4—C10	1.451 (4)	C7—H7C	0.9600
O1—C1	1.211 (3)	C8—H8	0.9300
O2—C5	1.205 (3)	C9—H9A	0.9600
O3—C5	1.310 (3)	C9—H9B	0.9600
O3—H3	0.8200	C9—H9C	0.9600
O4—C8	1.241 (3)	C10—H10A	0.9600
C1—C2	1.503 (4)	C10—H10B	0.9600
C2—C3	1.382 (4)	C10—H10C	0.9600
			
C2^i^—N1—C2	118.0 (3)	N3—C6—C7	126.3 (2)
C1—N2—N3	121.2 (2)	N3—C6—C5	117.3 (2)
C1—N2—H2	119.4	C7—C6—C5	116.4 (2)
N3—N2—H2	119.4	C6—C7—H7A	109.5
C6—N3—N2	115.1 (2)	C6—C7—H7B	109.5
C8—N4—C9	120.2 (3)	H7A—C7—H7B	109.5
C8—N4—C10	121.7 (3)	C6—C7—H7C	109.5
C9—N4—C10	118.0 (3)	H7A—C7—H7C	109.5
C5—O3—H3	109.5	H7B—C7—H7C	109.5
O1—C1—N2	124.0 (3)	O4—C8—N4	125.1 (3)
O1—C1—C2	123.0 (2)	O4—C8—H8	117.4
N2—C1—C2	113.0 (2)	N4—C8—H8	117.4
N1—C2—C3	122.8 (3)	N4—C9—H9A	109.5
N1—C2—C1	117.8 (2)	N4—C9—H9B	109.5
C3—C2—C1	119.4 (2)	H9A—C9—H9B	109.5
C4—C3—C2	118.3 (3)	N4—C9—H9C	109.5
C4—C3—H3A	120.9	H9A—C9—H9C	109.5
C2—C3—H3A	120.9	H9B—C9—H9C	109.5
C3—C4—C3^i^	119.9 (4)	N4—C10—H10A	109.5
C3—C4—H4	120.1	N4—C10—H10B	109.5
C3^i^—C4—H4	120.1	H10A—C10—H10B	109.5
O2—C5—O3	124.2 (3)	N4—C10—H10C	109.5
O2—C5—C6	120.3 (3)	H10A—C10—H10C	109.5
O3—C5—C6	115.5 (2)	H10B—C10—H10C	109.5

Symmetry codes: (i) −*x*+1, *y*, −*z*+3/2.

### Hydrogen-bond geometry (Å, °)

**Table:**

*D*—H···*A*	*D*—H	H···*A*	*D*···*A*	*D*—H···*A*
O3—H3···O4	0.82	1.75	2.574 (3)	178
